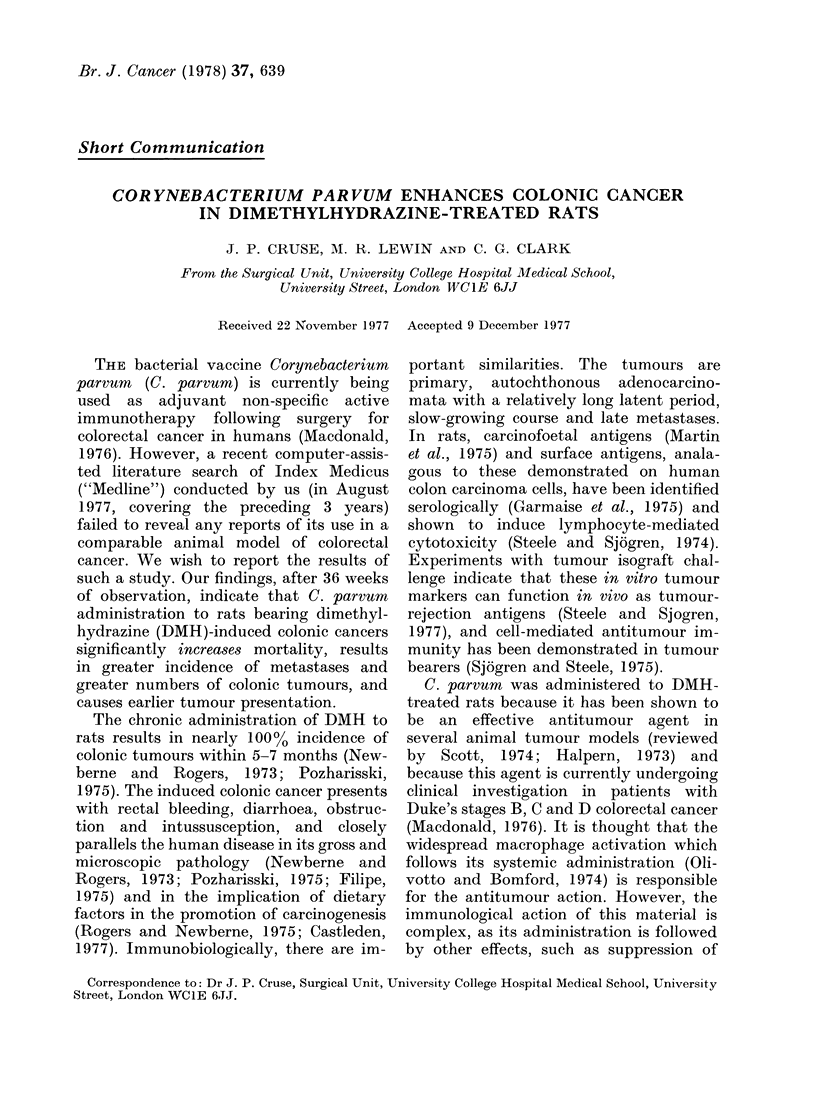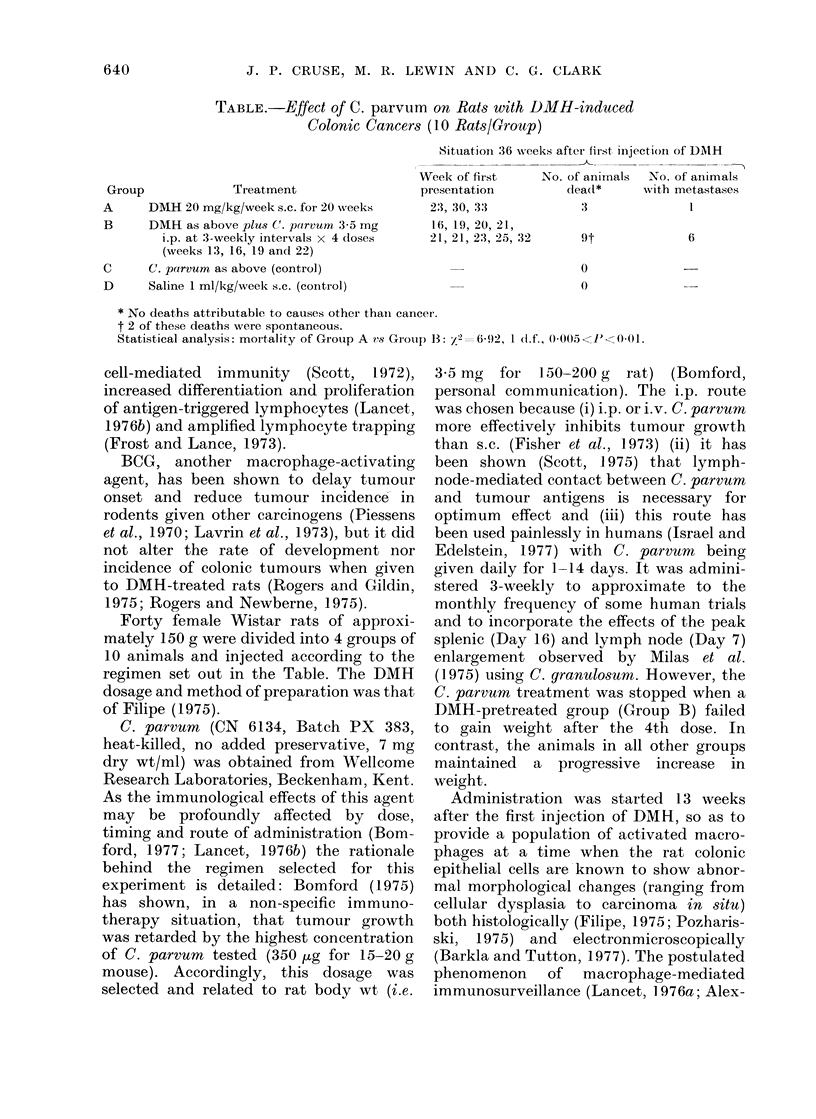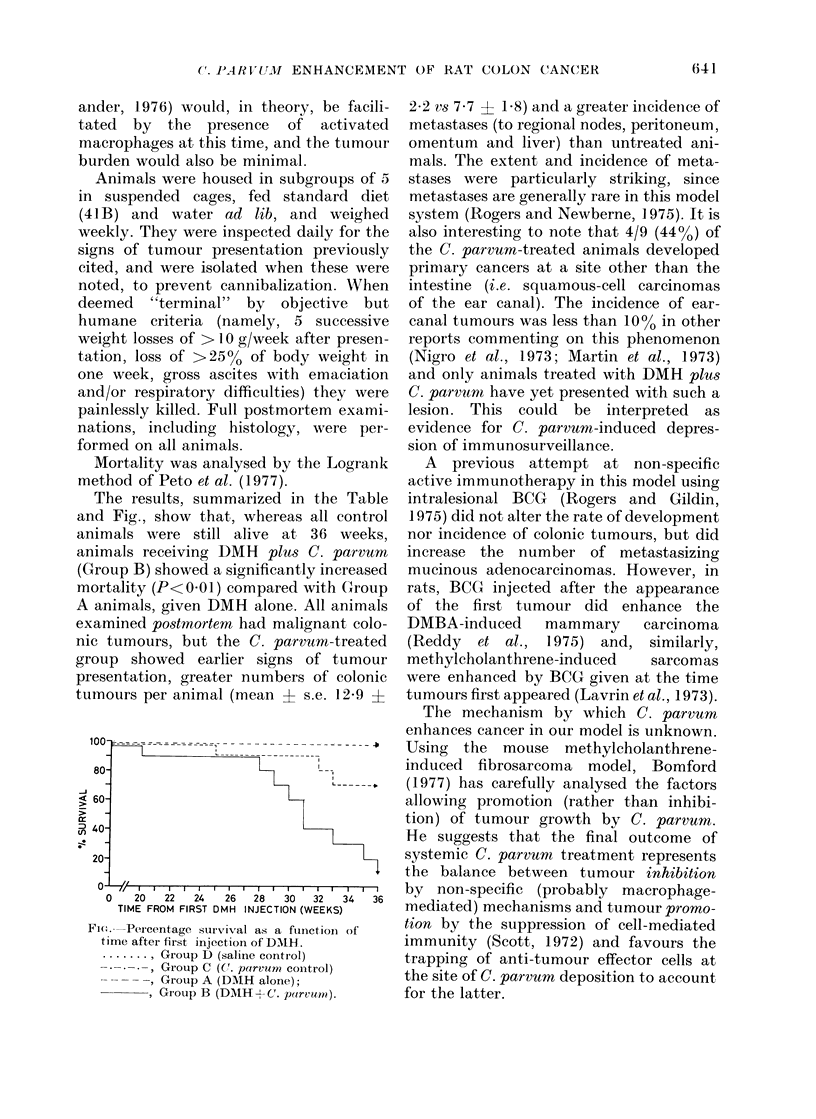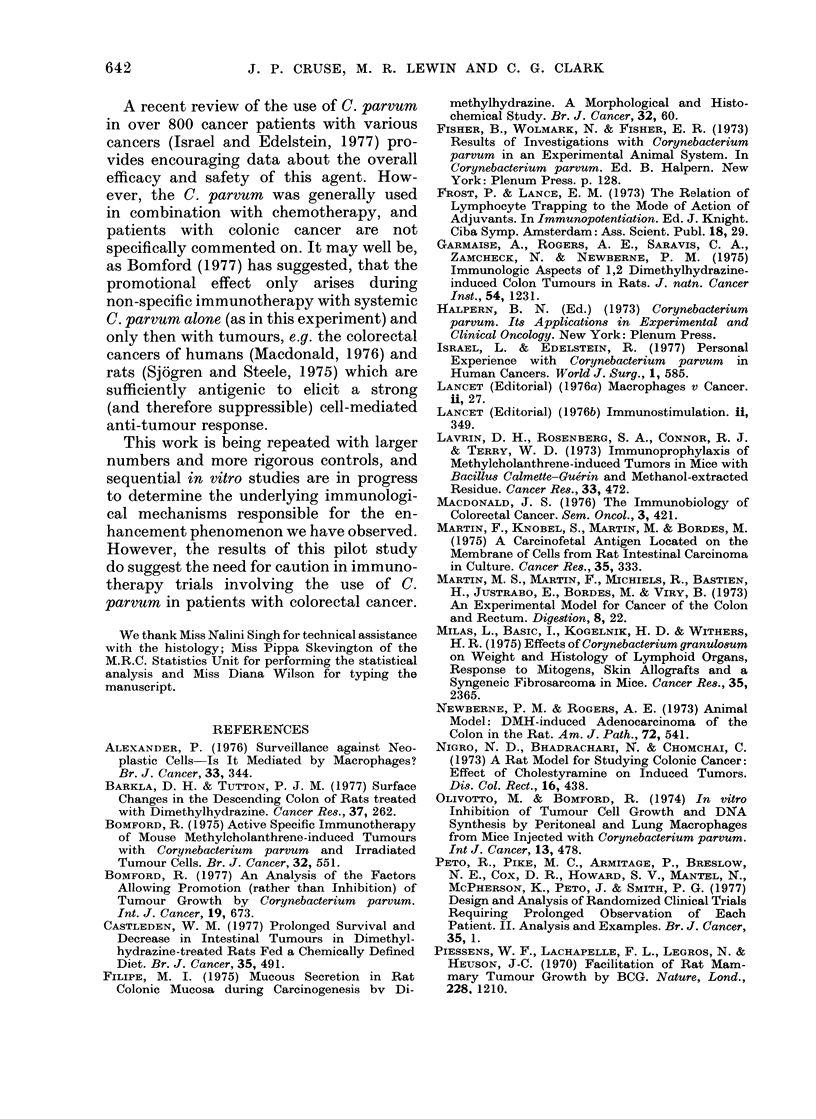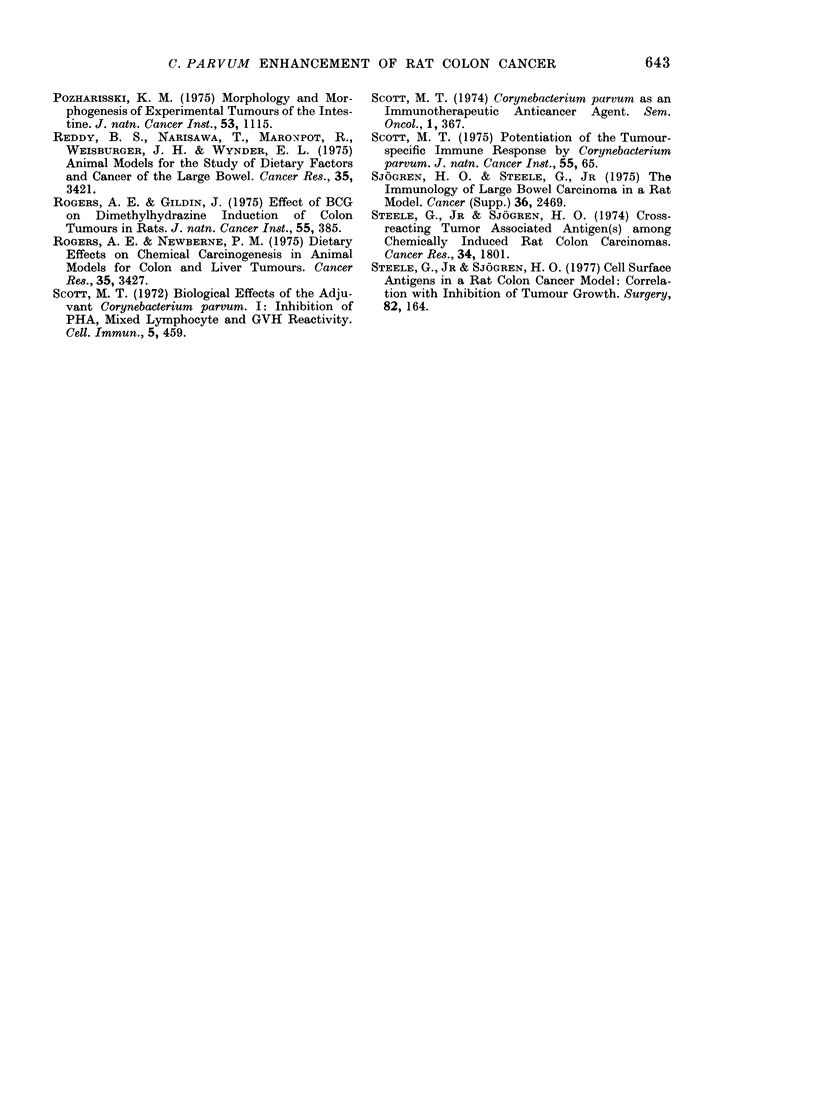# Corynebacterium parvum enhances colonic cancer in dimethylhydrazine-treated rats.

**DOI:** 10.1038/bjc.1978.94

**Published:** 1978-04

**Authors:** J. P. Cruse, M. R. Lewin, C. G. Clark


*Br. J. Cancer* (1978) **37**, 639

### *Short Communication*

### *CORYNEBACTERIUM PARVUM* ENHANCES COLONIC CANCER IN DIMETHYLHYDRAZINE-TREATED RATS

J. P. CRUSE, M. R. LEWIN AND C. G. CLARK

*From the Surgical Unit, University College Hospital Medical School, University Street, London WC1E 6JJ*

Received 22 November 1977 Accepted 9 December 1977

THE bacterial vaccine *Corynebacterium parvum* (*C. parvum*) is currently being used as adjuvant non-specific active immunotherapy following surgery for colorectal cancer in humans (Macdonald, 1976). However, a recent computer-assisted literature search of Index Medicus ("Medline") conducted by us (in August 1977, covering the preceding 3 years) failed to reveal any reports of its use in a comparable animal model of colorectal cancer. We wish to report the results of such a study. Our findings, after 36 weeks of observation, indicate that *C. parvum* administration to rats bearing dimethylhydrazine (DMH)-induced colonic cancers significantly *increases* mortality, results in greater incidence of metastases and greater numbers of colonic tumours, and causes earlier tumour presentation.

The chronic administration of DMH to rats results in nearly 100% incidence of colonic tumours within 5–7 months (Newberne and Rogers, 1973; Pozharisski, 1975). The induced colonic cancer presents with rectal bleeding, diarrhoea, obstruction and intussusception, and closely parallels the human disease in its gross and microscopic pathology (Newberne and Rogers, 1973; Pozharisski, 1975; Filipe, 1975) and in the implication of dietary factors in the promotion of carcinogenesis (Rogers and Newberne, 1975; Castleden, 1977). Immunobiologically, there are important similarities. The tumours are primary, autochthonous adenocarcinomata with a relatively long latent period, slow-growing course and late metastases. In rats, carcinofoetal antigens (Martin *et al.*, 1975) and surface antigens, analagous to these demonstrated on human colon carcinoma cells, have been identified serologically (Garmaise *et al.*, 1975) and shown to induce lymphocyte-mediated cytotoxicity (Steele and Sjögren, 1974). Experiments with tumour isograft challenge indicate that these *in vitro* tumour markers can function *in vivo* as tumour-rejection antigens (Steele and Sjogren, 1977), and cell-mediated antitumour immunity has been demonstrated in tumour bearers (Sjögren and Steele, 1975).

*C. parvum* was administered to DMH-treated rats because it has been shown to be an effective antitumour agent in several animal tumour models (reviewed by Scott, 1974; Halpern, 1973) and because this agent is currently undergoing clinical investigation in patients with Duke's stages B, C and D colorectal cancer (Macdonald, 1976). It is thought that the widespread macrophage activation which follows its systemic administration (Olivotto and Bomford, 1974) is responsible for the antitumour action. However, the immunological action of this material is complex, as its administration is followed by other effects, such as suppression of

Correspondence to: Dr J. P. Cruse, Surgical Unit, University College Hospital Medical School, University Street, London WC1E 6JJ.

TABLE.—*Effect of* C. parvum *on Rats with DMH-induced Colonic Cancers* (10 *Rats/Group*)

| Group | Treatment | Situation 36 weeks after first injection of DMH: Week of first presentation | No. of animals dead* | No. of animals with metastases |
|---|---|---|---|---|
| A | DMH 20 mg/kg/week s.c. for 20 weeks | 23, 30, 33 | 3 | 1 |
| B | DMH as above *plus C. parvum* 3·5 mg i.p. at 3-weekly intervals × 4 doses (weeks 13, 16, 19 and 22) | 16, 19, 20, 21, 21, 21, 23, 25, 32 | 9† | 6 |
| C | *C. parvum* as above (control) | — | 0 | — |
| D | Saline 1 ml/kg/week s.c. (control) | — | 0 | — |

* No deaths attributable to causes other than cancer.
† 2 of these deaths were spontaneous.
Statistical analysis: mortality of Group A *vs* Group B: $\chi^2 = 6 \cdot 92$, 1 d.f., $0 \cdot 005 < P < 0 \cdot 01$.

cell-mediated immunity (Scott, 1972), increased differentiation and proliferation of antigen-triggered lymphocytes (Lancet, 1976*b*) and amplified lymphocyte trapping (Frost and Lance, 1973).

BCG, another macrophage-activating agent, has been shown to delay tumour onset and reduce tumour incidence in rodents given other carcinogens (Piessens *et al.*, 1970; Lavrin *et al.*, 1973), but it did not alter the rate of development nor incidence of colonic tumours when given to DMH-treated rats (Rogers and Gildin, 1975; Rogers and Newberne, 1975).

Forty female Wistar rats of approximately 150 g were divided into 4 groups of 10 animals and injected according to the regimen set out in the Table. The DMH dosage and method of preparation was that of Filipe (1975).

*C. parvum* (CN 6134, Batch PX 383, heat-killed, no added preservative, 7 mg dry wt/ml) was obtained from Wellcome Research Laboratories, Beckenham, Kent. As the immunological effects of this agent may be profoundly affected by dose, timing and route of administration (Bomford, 1977; Lancet, 1976*b*) the rationale behind the regimen selected for this experiment is detailed: Bomford (1975) has shown, in a non-specific immunotherapy situation, that tumour growth was retarded by the highest concentration of *C. parvum* tested (350 μg for 15–20 g mouse). Accordingly, this dosage was selected and related to rat body wt (*i.e.* 3·5 mg for 150–200 g rat) (Bomford, personal communication). The i.p. route was chosen because (i) i.p. or i.v. *C. parvum* more effectively inhibits tumour growth than s.c. (Fisher *et al.*, 1973) (ii) it has been shown (Scott, 1975) that lymph-node-mediated contact between *C. parvum* and tumour antigens is necessary for optimum effect and (iii) this route has been used painlessly in humans (Israel and Edelstein, 1977) with *C. parvum* being given daily for 1–14 days. It was administered 3-weekly to approximate to the monthly frequency of some human trials and to incorporate the effects of the peak splenic (Day 16) and lymph node (Day 7) enlargement observed by Milas *et al.* (1975) using *C. granulosum*. However, the *C. parvum* treatment was stopped when a DMH-pretreated group (Group B) failed to gain weight after the 4th dose. In contrast, the animals in all other groups maintained a progressive increase in weight.

Administration was started 13 weeks after the first injection of DMH, so as to provide a population of activated macrophages at a time when the rat colonic epithelial cells are known to show abnormal morphological changes (ranging from cellular dysplasia to carcinoma *in situ*) both histologically (Filipe, 1975; Pozharisski, 1975) and electronmicroscopically (Barkla and Tutton, 1977). The postulated phenomenon of macrophage-mediated immunosurveillance (Lancet, 1976*a*; Alexander, 1976) would, in theory, be facilitated by the presence of activated macrophages at this time, and the tumour burden would also be minimal.

Animals were housed in subgroups of 5 in suspended cages, fed standard diet (41B) and water *ad lib*, and weighed weekly. They were inspected daily for the signs of tumour presentation previously cited, and were isolated when these were noted, to prevent cannibalization. When deemed "terminal" by objective but humane criteria (namely, 5 successive weight losses of $>10$ g/week after presentation, loss of $>25\%$ of body weight in one week, gross ascites with emaciation and/or respiratory difficulties) they were painlessly killed. Full postmortem examinations, including histology, were performed on all animals.

Mortality was analysed by the Logrank method of Peto *et al.* (1977).

The results, summarized in the Table and Fig., show that, whereas all control animals were still alive at 36 weeks, animals receiving DMH *plus C. parvum* (Group B) showed a significantly increased mortality ($P<0{\cdot}01$) compared with Group A animals, given DMH alone. All animals examined *postmortem* had malignant colonic tumours, but the *C. parvum*-treated group showed earlier signs of tumour presentation, greater numbers of colonic tumours per animal (mean $\pm$ s.e. $12{\cdot}9 \pm 2{\cdot}2$ *vs* $7{\cdot}7 \pm 1{\cdot}8$) and a greater incidence of metastases (to regional nodes, peritoneum, omentum and liver) than untreated animals. The extent and incidence of metastases were particularly striking, since metastases are generally rare in this model system (Rogers and Newberne, 1975). It is also interesting to note that 4/9 (44%) of the *C. parvum*-treated animals developed primary cancers at a site other than the intestine (*i.e.* squamous-cell carcinomas of the ear canal). The incidence of ear-canal tumours was less than 10% in other reports commenting on this phenomenon (Nigro *et al.*, 1973; Martin *et al.*, 1973) and only animals treated with DMH *plus C. parvum* have yet presented with such a lesion. This could be interpreted as evidence for *C. parvum*-induced depression of immunosurveillance.

FIG.—Percentage survival as a function of time after first injection of DMH.

·······, Group D (saline control)

–·–·–·–, Group C (*C. parvum* control)

– – – – –, Group A (DMH alone);

————, Group B (DMH + *C. parvum*).

A previous attempt at non-specific active immunotherapy in this model using intralesional BCG (Rogers and Gildin, 1975) did not alter the rate of development nor incidence of colonic tumours, but did increase the number of metastasizing mucinous adenocarcinomas. However, in rats, BCG injected after the appearance of the first tumour did enhance the DMBA-induced mammary carcinoma (Reddy *et al.*, 1975) and, similarly, methylcholanthrene-induced sarcomas were enhanced by BCG given at the time tumours first appeared (Lavrin *et al.*, 1973).

The mechanism by which *C. parvum* enhances cancer in our model is unknown. Using the mouse methylcholanthrene-induced fibrosarcoma model, Bomford (1977) has carefully analysed the factors allowing promotion (rather than inhibition) of tumour growth by *C. parvum*. He suggests that the final outcome of systemic *C. parvum* treatment represents the balance between tumour *inhibition* by non-specific (probably macrophage-mediated) mechanisms and tumour *promotion* by the suppression of cell-mediated immunity (Scott, 1972) and favours the trapping of anti-tumour effector cells at the site of *C. parvum* deposition to account for the latter.

A recent review of the use of *C. parvum* in over 800 cancer patients with various cancers (Israel and Edelstein, 1977) provides encouraging data about the overall efficacy and safety of this agent. However, the *C. parvum* was generally used in combination with chemotherapy, and patients with colonic cancer are not specifically commented on. It may well be, as Bomford (1977) has suggested, that the promotional effect only arises during non-specific immunotherapy with systemic *C. parvum alone* (as in this experiment) and only then with tumours, *e.g.* the colorectal cancers of humans (Macdonald, 1976) and rats (Sjögren and Steele, 1975) which are sufficiently antigenic to elicit a strong (and therefore suppressible) cell-mediated anti-tumour response.

This work is being repeated with larger numbers and more rigorous controls, and sequential *in vitro* studies are in progress to determine the underlying immunological mechanisms responsible for the enhancement phenomenon we have observed. However, the results of this pilot study do suggest the need for caution in immunotherapy trials involving the use of *C. parvum* in patients with colorectal cancer.

We thank Miss Nalini Singh for technical assistance with the histology; Miss Pippa Skevington of the M.R.C. Statistics Unit for performing the statistical analysis and Miss Diana Wilson for typing the manuscript.

### REFERENCES

ALEXANDER, P. (1976) Surveillance against Neoplastic Cells—Is It Mediated by Macrophages? *Br. J. Cancer*, **33**, 344.

BARKLA, D. H. & TUTTON, P. J. M. (1977) Surface Changes in the Descending Colon of Rats treated with Dimethylhydrazine. *Cancer Res.*, **37**, 262.

BOMFORD, R. (1975) Active Specific Immunotherapy of Mouse Methylcholanthrene-induced Tumours with *Corynebacterium parvum* and Irradiated Tumour Cells. *Br. J. Cancer*, **32**, 551.

BOMFORD, R. (1977) An Analysis of the Factors Allowing Promotion (rather than Inhibition) of Tumour Growth by *Corynebacterium parvum*. *Int. J. Cancer*, **19**, 673.

CASTLEDEN, W. M. (1977) Prolonged Survival and Decrease in Intestinal Tumours in Dimethylhydrazine-treated Rats Fed a Chemically Defined Diet. *Br. J. Cancer*, **35**, 491.

FILIPE, M. I. (1975) Mucous Secretion in Rat Colonic Mucosa during Carcinogenesis bv Dimethylhydrazine. A Morphological and Histochemical Study. *Br. J. Cancer*, **32**, 60.

FISHER, B., WOLMARK, N. & FISHER, E. R. (1973) Results of Investigations with *Corynebacterium parvum* in an Experimental Animal System. In *Corynebacterium parvum*. Ed. B. Halpern. New York: Plenum Press. p. 128.

FROST, P. & LANCE, E. M. (1973) The Relation of Lymphocyte Trapping to the Mode of Action of Adjuvants. In *Immunopotentiation*. Ed. J. Knight. Ciba Symp. Amsterdam: Ass. Scient. Publ. **18**, 29.

GARMAISE, A., ROGERS, A. E., SARAVIS, C. A., ZAMCHECK, N. & NEWBERNE, P. M. (1975) Immunologic Aspects of 1,2 Dimethylhydrazine-induced Colon Tumours in Rats. *J. natn. Cancer Inst.*, **54**, 1231.

HALPERN, B. N. (Ed.) (1973) *Corynebacterium parvum. Its Applications in Experimental and Clinical Oncology*. New York: Plenum Press.

ISRAEL, L. & EDELSTEIN, R. (1977) Personal Experience with *Corynebacterium parvum* in Human Cancers. *World J. Surg.*, **1**, 585.

LANCET (Editorial) (1976*a*) Macrophages *v* Cancer. **ii**, 27.

LANCET (Editorial) (1976*b*) Immunostimulation. **ii**, 349.

LAVRIN, D. H., ROSENBERG, S. A., CONNOR, R. J. & TERRY, W. D. (1973) Immunoprophylaxis of Methylcholanthrene-induced Tumors in Mice with *Bacillus Calmette–Guérin* and Methanol-extracted Residue. *Cancer Res.*, **33**, 472.

MACDONALD, J. S. (1976) The Immunobiology of Colorectal Cancer. *Sem. Oncol.*, **3**, 421.

MARTIN, F., KNOBEL, S., MARTIN, M. & BORDES, M. (1975) A Carcinofetal Antigen Located on the Membrane of Cells from Rat Intestinal Carcinoma in Culture. *Cancer Res.*, **35**, 333.

MARTIN, M. S., MARTIN, F., MICHIELS, R., BASTIEN, H., JUSTRABO, E., BORDES, M. & VIRY, B. (1973) An Experimental Model for Cancer of the Colon and Rectum. *Digestion*, **8**, 22.

MILAS, L., BASIC, I., KOGELNIK, H. D. & WITHERS, H. R. (1975) Effects of *Corynebacterium granulosum* on Weight and Histology of Lymphoid Organs, Response to Mitogens, Skin Allografts and a Syngeneic Fibrosarcoma in Mice. *Cancer Res.*, **35**, 2365.

NEWBERNE, P. M. & ROGERS, A. E. (1973) Animal Model: DMH-induced Adenocarcinoma of the Colon in the Rat. *Am. J. Path.*, **72**, 541.

NIGRO, N. D., BHADRACHARI, N. & CHOMCHAI, C. (1973) A Rat Model for Studying Colonic Cancer: Effect of Cholestyramine on Induced Tumors. *Dis. Col. Rect.*, **16**, 438.

OLIVOTTO, M. & BOMFORD, R. (1974) *In vitro* Inhibition of Tumour Cell Growth and DNA Synthesis by Peritoneal and Lung Macrophages from Mice Injected with *Corynebacterium parvum*. *Int J. Cancer*, **13**, 478.

PETO, R., PIKE, M. C., ARMITAGE, P., BRESLOW, N. E., COX, D. R., HOWARD, S. V., MANTEL, N., MCPHERSON, K., PETO, J. & SMITH, P. G. (1977) Design and Analysis of Randomized Clinical Trials Requiring Prolonged Observation of Each Patient. II. Analysis and Examples. *Br. J. Cancer*, **35**, 1.

PIESSENS, W. F., LACHAPELLE, F. L., LEGROS, N. & HEUSON, J-C. (1970) Facilitation of Rat Mammary Tumour Growth by BCG. *Nature, Lond.*, **228**, 1210.

POZHARISSKI, K. M. (1975) Morphology and Morphogenesis of Experimental Tumours of the Intestine. *J. natn. Cancer Inst.*, **53**, 1115.

REDDY, B. S., NARISAWA, T., MARONPOT, R., WEISBURGER, J. H. & WYNDER, E. L. (1975) Animal Models for the Study of Dietary Factors and Cancer of the Large Bowel. *Cancer Res.*, **35**, 3421.

ROGERS, A. E. & GILDIN, J. (1975) Effect of BCG on Dimethylhydrazine Induction of Colon Tumours in Rats. *J. natn. Cancer Inst.*, **55**, 385.

ROGERS, A. E. & NEWBERNE, P. M. (1975) Dietary Effects on Chemical Carcinogenesis in Animal Models for Colon and Liver Tumours. *Cancer Res.*, **35**, 3427.

SCOTT, M. T. (1972) Biological Effects of the Adjuvant *Corynebacterium parvum*. I: Inhibition of PHA, Mixed Lymphocyte and GVH Reactivity. *Cell. Immun.*, **5**, 459.

SCOTT, M. T. (1974) *Corynebacterium parvum* as an Immunotherapeutic Anticancer Agent. *Sem. Oncol.*, **1**, 367.

SCOTT, M. T. (1975) Potentiation of the Tumour-specific Immune Response by *Corynebacterium parvum*. *J. natn. Cancer Inst.*, **55**, 65.

SJÖGREN, H. O. & STEELE, G., JR (1975) The Immunology of Large Bowel Carcinoma in a Rat Model. *Cancer* (Supp.) **36**, 2469.

STEELE, G., JR & SJÖGREN, H. O. (1974) Cross-reacting Tumor Associated Antigen(s) among Chemically Induced Rat Colon Carcinomas. *Cancer Res.*, **34**, 1801.

STEELE, G., JR & SJÖGREN, H. O. (1977) Cell Surface Antigens in a Rat Colon Cancer Model: Correlation with Inhibition of Tumour Growth. *Surgery*, **82**, 164.